# Educational interventions need evidence too. Commentary: A crisis in comparative psychology: where have all the undergraduates gone?

**DOI:** 10.3389/fpsyg.2015.01868

**Published:** 2015-12-02

**Authors:** Lewis G. Dean

**Affiliations:** School of Psychology and Neuroscience, University of St AndrewsSt Andrews, UK

**Keywords:** comparative psychology, teaching and learning, public engagement, undergraduate students, science education

Abramson laments that there is a crisis in comparative psychology, measuring success by recruitment to undergraduate courses. By employing such a narrow perspective on how to measure the success of a field, the article offers a narrow viewpoint on the issue and, as such, I am concerned that the solutions it offers may be ineffective. I urge academics to look outwards and work with other groups, such as the students themselves. If we believe our field is “in crisis,” isolating ourselves is not a solution.

The target article focuses on the recruitment of undergraduate students to study comparative psychology. At a time when research outputs are most often used to measure individual academics (Kelly and Jennions, 2006; McGrail et al., 2006), Abramson highlights students as an integral part of any academic field. I am concerned, however, that deeming an academic field “in crisis” based solely on one measure—the number of undergraduates on one very narrowly defined undergraduate course—is not a robust measure.

There are a number of solutions offered by Abramson in the article to increase undergraduate recruitment to comparative psychology. It is noteworthy that none of them advocate engaging *with* any of the groups that are mentioned in an inclusive and open manner. Students should be “urged” to study certain courses, public audiences should be “taught” about the topic. What is absent is the suggestion that these groups are integral to finding a solution. There is no suggestion that we should find out why undergraduate students are picking certain modules or whether school leavers selecting psychology as a university course are aware of comparative research in psychology. I am concerned that without accurate information on the causes, we cannot start to devise suitable solutions.

Research by sociologists studying the university choices of school children suggests that it is a complex decision making process. For example, Holmegaard et al. (2014) show that the choice of subject studied at university is not a isolated, single decision, but rather is an ongoing process, influenced by a range of factors. The subject choices of students may depend on factors including which subjects they enjoy at school, their socio-economic background, and the opinions of friends and family (Brooks, 2003; Purcell et al., 2008; Holmegaard et al., 2014). If we are to come up with a range of solutions to the crisis in student recruitment diagnosed by Abramson, we should base them on the best available evidence.

There is no evidence presented that the solutions put forward by Abramson will have a significant effect on the problem that he has diagnosed. There is no discussion of why studying, for example, comparative literature will make students more predisposed to take up comparative psychology. Indeed, we have no idea that these solutions are actually addressing the concerns of students who have chosen not to take comparative psychology. The only evidence that is cited is an informal survey of students on a comparative psychology course, which suggests that they do not define the subject in the same way as the author. It is unclear whether students' choices (both those that opt to take the course and those that don't) would have changed had their definition of comparative psychology been different. Without evidence that this is a problem in the minds of students who are avoiding the course we don't know whether the proposed solution addresses the root cause of the problem.

If we define comparative psychology as narrowly as Abramson (see McMillan and Sturdy, 2015 for a discussion on this) and perceive that there is a problem with undergraduate recruitment then inventing solutions cannot be enough. We need to research what the perceptions of comparative psychology are in the minds of students and prospective students, and what barriers there are to them taking courses in the topic. The students are not empty vessels that need information from a professor to correct their opinions (the so-called “information deficit model”; Gregory and Miller, 2000). Public engagement with science has seen a move away from this deficit approach to one of collaboration, where scientists and public audiences come together to explore topics, identify differences in opinion and discover more about a topic in a manner than benefits both parties precisely because the deficit model was found to be an ineffective way of engaging audiences with scientific research (Irwin, 2014).

By ignoring the need for evidence, we do not know whether Abramson has addressed the correct questions when formulating his solutions, much less whether they will be successful interventions. If there are too few comparative psychology undergraduates and if this is going to be to the detriment of the field in years to come then I agree that we should find solutions to it. We are not, however, going to find lasting solutions by unilaterally designing them without any recourse to evidence. If we are really concerned about undergraduates, we should take care not to treat them as numbers to simply be manipulated. We should engage with and work alongside them to find appropriate solutions.

## Funding

LD is supported by a grant from the John Templeton Foundation ID 40128 to Professors Andrew Whiten and Kevin Laland.

### Conflict of interest statement

The author declares that the research was conducted in the absence of any commercial or financial relationships that could be construed as a potential conflict of interest.
